# NADH Dehydrogenase Subunit-2 237 Leu/Met Polymorphism Influences the Association of Coffee Consumption with Serum Chloride Levels in Male Japanese Health Checkup Examinees: An Exploratory Cross-Sectional Analysis

**DOI:** 10.3390/nu10101344

**Published:** 2018-09-20

**Authors:** Akatsuki Kokaze, Mamoru Ishikawa, Naomi Matsunaga, Kanae Karita, Masao Yoshida, Hirotaka Ochiai, Takako Shirasawa, Takahiko Yoshimoto, Akira Minoura, Kosuke Oikawa, Masao Satoh, Hiromi Hoshino, Yutaka Takashima

**Affiliations:** 1 Department of Hygiene, Public Health and Preventive Medicine, Showa University School of Medicine, 1-5-8 Hatanodai, Shinagawa-ku, Tokyo 142-8555, Japan; h-ochiai@med.showa-u.ac.jp (H.O.); shirasawa@med.showa-u.ac.jp (T.S.); yoshimotot@med.showa-u.ac.jp (T.Y.); minoaki@med.showa-u.ac.jp (A.M.); k-oikawa@med.showa-u.ac.jp (K.O.); hhiromi@med.showa-u.ac.jp (H.H.); 2 Department of Public Health, Kyorin University School of Medicine, 6-20-2 Shinkawa, Mitaka-shi, Tokyo 181-8611, Japan; ishikawa-m@genkiplaze.or.jp (M.I.); naomim@ks.kyorin-u.ac.jp (N.M.); kanae@ks.kyorin-u.ac.jp (K.K.); yohhy@ks.kyorin-u.ac.jp (M.Y.); yutakat@kyorin-u.ac.jp (Y.T.); 3 Mito Red Cross Hospital, 3-12-48 Sannomaru, Mito-shi, Ibaraki 310-0011, Japan; 4 School of Medical Technology and Health, Faculty of Health and Medical Care, Saitama Medical University, 1397-1 Yamane, Hidaka-shi, Saitama 350-1241, Japan; satoma@saitama-med.ac.jp

**Keywords:** cardiovascular disease, coffee consumption, gene-diet interaction, longevity, NADH dehydrogenase, polymorphism, serum chloride levels

## Abstract

Background: Nicotinamide adenine dinucleotide (NADH) dehydrogenase subunit-2 237 leucine/methionine (ND2-237 Leu/Met) polymorphism has been shown to modify the association of coffee consumption with the risk of hypertension, dyslipidemia, and abnormal glucose tolerance, and low serum chloride levels have been shown to be associated with all-cause and cardiovascular disease mortality. Therefore, the purpose of the present study was to investigate whether ND2-237 Leu/Met polymorphism influences the association of coffee consumption with serum chloride levels in male Japanese health checkup examinees. Methods: From among individuals visiting the hospital for a regular medical checkup, 402 men (mean age ± standard deviation, 53.9 ± 7.8 years) were selected for inclusion in the study. After ND2-237 Leu/Met genotyping, we conducted an exploratory cross-sectional study to examine the combined association of ND2-237 Leu/Met polymorphism and coffee consumption with serum electrolyte levels. Results: After adjusting for age, body mass index, habitual smoking, alcohol consumption, green tea consumption, and antihypertensive medication, coffee consumption significantly increased serum chloride levels (*p* for trend = 0.001) in men with the ND2-237Leu genotype. After these adjustments, the odds ratios (ORs) for low levels of serum chloride, defined as <100 mEq/L, were found to be dependent on coffee consumption (*p* for trend = 0.001). In addition, the OR for low levels of serum chloride was significantly lower in men with the ND2-237Leu genotype who consumed ≥4 compared with <1 cup of coffee per day (OR = 0.096, 95% confidence interval = 0.010–0.934; *p* = 0.044). However, neither serum chloride levels nor risk of low levels of serum chloride appeared to be dependent on coffee consumption. Conclusions: The results suggest that ND2-237 Leu/Met polymorphism modifies the association of coffee consumption with serum chloride levels in middle-aged Japanese men.

## 1. Background

Coffee intake has been shown to be a favorable health behavior [1,2,3], and habitual consumption (3–4 cups per day) is more likely to benefit than to harm health [3]. Several recent meta-analyses of prospective studies have reported finding an inverse relationship between habitual coffee consumption and both all-cause [3,4,5,6] and cardiovascular disease (CVD) [4,5] mortality. In addition, Rodríguez-Artalejo and López-García reported that habitual consumption of 3–5 cups of coffee per day reduces the risk of CVD by 15%, and that habitual consumption of >3–5 cups per day does not elevate the risk of CVD [7].

A population-based cohort study reported finding an association between lower serum chloride levels and both all-cause and CVD mortality [8]. That epidemiological study found that the risk ratio for CVD mortality associated with low serum chloride levels was equivalent to or higher than that observed for well-known risk factors of CVD, including hypertension, diabetes, and habitual smoking [8]. Furthermore, low levels of serum chloride have been reported to be associated with increased mortality and risk of CVD in patients with pre-dialysis chronic kidney disease [9], and serum chloride levels have been shown to be negatively associated with mortality in patients with a history of heart failure [10,11]. By contrast, in a large-scale follow-up study involving hypertensive adults, a 1 mEq/L increase in serum chloride levels was found to reduce all-cause mortality by 1.5% after adjusting for confounding factors [12].

Nicotinamide adenine dinucleotide (NADH) dehydrogenase subunit-2 237 leucine/methionine (ND2-237 Leu/Met) polymorphism has been reported to be associated with longevity in the Japanese population [13]. The frequency of the ND2-237Met genotype has been found to be substantially higher in Japanese centenarians compared with the general Japanese population [13], and individuals with the ND2-237Met genotype have been shown to be less likely than those with the ND2-237Leu genotype to develop lifestyle-related diseases [14,15,16,17,18].

ND2-237 Leu/Met polymorphism has been reported to modify the effect of coffee consumption on the risks of hypertension [19], glucose tolerance abnormality [20], dyslipidemia [21], liver damage [22], and anemia [23]. We previously reported that serum chloride levels were significantly lower in obese men with the ND2-237Met genotype than in those with the ND2-237Leu genotype [24]. However, to our knowledge, no studies have been conducted to investigate the joint association of ND2-237 Leu/Met polymorphism and coffee consumption with serum chloride levels.

Therefore, the purpose of the present exploratory cross-sectional study was to investigate whether ND2-237 Leu/Met polymorphism modifies the association of coffee consumption with serum chloride levels in male Japanese health checkup examinees.

## 2. Subjects and Methods

### 2.1. Study Participants

The study participants were recruited from among individuals visiting the Mito Red Cross Hospital for a regular medical checkup between August 1999 and August 2000. This study was conducted in accordance with the Declaration of Helsinki. The Ethics Committee of Kyorin University School of Medicine approved the study protocol. Written informed consent was obtained from all 602 volunteers before participation. Because of the insufficient number of women available for categorization into groups based on the ND2-237 Leu/Met genotype and coffee consumption, females were excluded, as were males with unclear or incomplete data. Finally, 402 Japanese men (mean age ± standard deviation, 53.9 ± 7.8 years) were included in the analysis.

### 2.2. Data Collection

#### 2.2.1. Clinical Measurements

The participants’ anthropometric, biophysical, and biochemical data were obtained from the results of regular medical checkups. Serum electrolyte levels—namely serum sodium, chloride, potassium, or calcium levels—were determined using an auto-analyzer (HITACHI 7600-110S; Hitachi High Technology Corp., Tokyo, Japan). Body mass index (BMI) was calculated as weight (kg) divided by the square of height (m). Information on antihypertensive treatment was derived from the participants’ health records.

#### 2.2.2. Self-Administered Questionnaire

A survey regarding coffee intake, habitual smoking, alcohol consumption, and green tea intake was conducted on the participants via a self-administered questionnaire. Similar to previous reports [19,20,21,22,23], coffee consumption was categorized based on the number of cups of coffee consumed per day (<1, 1–3, and ≥4 cups). Habitual smoking was categorized as non- or ex-smokers and current smokers. Alcohol consumption was categorized based on drinking frequency (daily drinkers; occasional drinkers [those who drink several times per week or month]; and non- or ex-drinkers). Green tea consumption was categorized based on the number of cups of green tea consumed per day (≤1, 2-4, and ≥5 cups).

### 2.3. Genotyping

ND2-237 genotyping methods have been described previously [23]. Briefly, DNA was extracted from white blood cells. ND2-237 Leu/Met genotype was determined using the polymerase chain reaction-restriction fragment length polymorphism test. The absence or presence of an *Alu*I site was designated as ND2-237Met or ND2-237Leu, respectively.

### 2.4. Statistical Analyses

All statistical analyses were performed using SAS statistical software (version 9.2 for Windows; SAS Institute, Inc., Cary, NC, USA). Multiple logistic regression analysis was conducted to calculate odds ratios (ORs) for the risk of low levels of serum chloride. In accordance with previous epidemiological studies [8,12], low levels of serum chloride were defined as <100 mEq/L. For the multiple logistic regression analysis and analysis of covariance, habitual smoking (non- or ex-smokers = 0, current smokers = 1), alcohol consumption (non- or ex-drinkers = 0, occasional drinkers = 1, daily drinkers = 2), green tea consumption (≤1 cup per day = 1, 2–4 cups per day = 2, ≥5 cups per day = 3) and antihypertensive treatment (not receiving antihypertensive treatment = 0, receiving antihypertensive treatment = 1) were numerically coded. Two-tailed *p* values <0.05 were considered statistically significant.

## 3. Results

No statistically significant differences in serum electrolyte levels—namely serum sodium, chloride, potassium, or calcium levels—were observed between the ND2-237Leu and ND2-237Met genotypes (Table 1).

A significant positive association was observed between coffee consumption and serum chloride levels in the men with the ND2-237Leu genotype (*p* for trend = 0.001) (Table 2). Moreover, serum chloride levels were significantly higher in the participants who consumed ≥4 compared with <1 or 1–3 cups of coffee per day (*p* = 0.001 and *p* = 0.026, respectively). After adjusting for age, BMI, habitual smoking, alcohol consumption, green tea consumption, and antihypertensive medication, a significant positive association was found between coffee consumption and serum chloride levels (*p* for trend = 0.002). In addition, serum chloride levels were significantly higher in the participants who consumed ≥4 compared with <1 cup of coffee per day (*p* = 0.010). Moreover, a significant positive association was observed between coffee consumption and serum sodium levels in men with the ND2-237Leu genotype (*p* for trend = 0.033); a significant positive association was also found between coffee consumption and serum sodium levels (*p* for trend = 0.044). By contrast, no statistically significant association was observed between coffee consumption and serum electrolyte levels in the men with the ND2-237Met genotype.

A significant negative association was observed between coffee consumption and the risk of low levels of serum chloride among men with the ND2-237Leu genotype (*p* for trend = 0.032) (Table 3). Moreover, the *OR* for low levels of serum chloride was significantly lower among men with the ND2-237Leu genotype who consumed ≥4 compared with <1 cup of coffee per day (*OR* = 0.125, 95% confidence interval [CI] = 0.016–0.973; *p* = 0.047). After adjusting for age, BMI, habitual smoking, alcohol consumption, green tea consumption, and antihypertensive medication, the risk of low levels of serum chloride was found to be dependent on coffee consumption (*p* for trend = 0.028). In addition, the *OR* for low levels of serum chloride was found to be significantly lower for men with the ND2-237Leu genotype who consumed ≥4 compared with <1 cup of coffee per day (*OR* = 0.096, 95% CI = 0.010–0.934; *p* = 0.044). However, the association between the ND2-237Met genotype and the risk of low levels of serum chloride did not appear to be statistically dependent on the amount of daily coffee consumption.

## 4. Discussion

Although somewhat limited by the small sample size, the results of the present study suggest that ND2-237 Leu/Met polymorphism and coffee consumption exert a joint association with serum chloride levels in middle-age male Japanese health checkup examinees; namely, serum chloride levels were positively associated with the amount of daily coffee consumption in men with the ND2-237Leu genotype. Moreover, the risk of low levels of serum chloride, which has been shown to be associated with a higher risk of CVD and mortality [8,9,10,11,12], was significantly lower in men with the ND2-237Leu genotype who consumed ≥4 compared with <1 cup of coffee per day. However, coffee consumption did not appear to affect serum chloride levels in men with the ND2-237Met genotype.

In regard to the role of the interplay between caffeine and mitochondria in the cardiovascular system, Ale-Agha, et al. recently established experimentally that caffeine acting jointly with mitochondrial cyclin-dependent kinase inhibitor 1B exerts protective effects against CVD [25]. However, although likely related to biochemical differences between ND2-237Leu and ND2-237Met in response to some compounds in coffee, the physiological mechanisms underlying the joint association of ND2-237 Leu/Met polymorphism and coffee consumption with serum chloride levels remains unknown. NADH dehydrogenase is known as a major site of the generation of reactive oxygen species (ROS) in mitochondria; it is also known itself to be a target of attack by ROS [26]. Some animal experiments have demonstrated that ND2-237Met protects NADH dehydrogenase from ROS attack and/or suppresses ROS production [27,28]. Moreover, coffee consumption has been shown to exert antioxidant potential in humans [29]. The results of our previous research suggest that moderate coffee intake exerts antioxidant effects in men with the ND2-237Leu but not the ND2-237Met genotype [19,20,22]. To elucidate the mechanisms underlying the joint association of ND2-237 Leu/Met polymorphism and coffee consumption with serum chloride levels, further biophysical and biochemical investigations are needed.

Previous cross-sectional studies have reported finding an interactive association of ND2-237 Leu/Met polymorphism and coffee consumption with the risks of hypertension [19], glucose tolerance abnormality [20], dyslipidemia [21], liver damage [22], and erythrocytic parameters [23]. The risk of hypertension was significantly lower in men with the ND2-237Leu genotype who consumed ≥2 compared with ≤1 cup of coffee per day [19], and that of glucose tolerance abnormality was also significantly lower in those who consumed ≥4 compared with <1 cup of coffee per day [20]. Consequently, the risk of abnormally elevated levels of serum liver enzymes was significantly lower in men who consumed ≥3 compared with <1 cup of coffee per day [22]. However, the risk of anemia was significantly higher in those who consumed ≥4 compared with <1 cup of coffee per day [23]. Meanwhile, the risk of dyslipidemia was significantly higher among men with the ND2-237Met genotype who consumed ≥1 compared with <1 cup of coffee per day [21]. Taken together, other than the risk of anemia, moderate levels of coffee intake appear to be more beneficial for health in men with the ND2-237Leu compared with the ND2-237Met genotype.

Although the focus of the present study was on serum chloride levels, serum sodium levels were also significantly and positively associated with coffee consumption in men with the ND2-237Leu genotype. In addition, in a population-based cohort study, De Bacquer, et al. reported finding a remarkably positive association between serum chloride and serum sodium levels [8]. In patients with acute decompensated heart failure, compared with that of serum chloride levels, the prognostic value of serum sodium levels was diminished [10]. Moreover, in patients with post-myocardial infarction accompanied by systolic dysfunction and heart failure, low levels of serum chloride were found to be associated with CVD mortality accompanied by low levels of serum sodium [30]. Recently, a large-scale follow-up study reported finding an association between low serum sodium levels and an increased risk of CVD mortality [31]; therefore, serum sodium levels may affect the pathophysiology of serum chloride levels for CVD. However, to verify whether serum chloride levels act jointly with serum sodium levels in regard to CVD in the general population, further epidemiological studies are needed.

A potential contradiction may exist in relation to these findings. Although low serum chloride levels have been shown to be associated with an increased risk of both all-cause and CVD mortality [8,9,10,11,12], the results of our previous study showed that serum chloride levels were significantly lower in men who had the ND2-237Met genotype—who have been reported to have a genetic tendency toward longevity [13] and resistance against life-threatening diseases [14,15,16,17,18]—compared with those with the ND2-237Leu genotype [24]. Therefore, the genetic advantage of having the ND2-237Met genotype may surpass the physiological disadvantage of having low serum chloride levels.

The present study did have several important limitations. First, the data were collected 18 years ago. Second, the sample size was relatively small. Third, the participants consisted of only men. Fourth, only a single population was analyzed; to prevent errors in genetic epidemiological studies, several independent data sets need to be analyzed. Fifth, although cross-sectional studies can suggest causal associations, they cannot prove valid causality. To overcome this limitation, a follow-up study involving a larger study sample that includes multiple populations is needed. Sixth, we did not obtain any data on salt intake or other dietary factors. Although no significant associations have been found between serum chloride levels and dietary sodium intake [32], higher sodium intake has been found to be a risk factor for CVD in Japan [33]. Potential correlations between additional dietary factors and coffee consumption have been reported [34]. Therefore, a food frequency questionnaire survey will be required in future studies. Finally, we based the categorization of habitual coffee consumption on the number of cups consumed per day. Whether any interaction exists between ND2-237 Leu/Met polymorphism and levels of caffeine, chlorogenic acids, or other unknown compounds in coffee on serum chloride levels remains unclear and therefore warrants further investigation.

## 5. Conclusions

The results of the present exploratory cross-sectional analysis suggest a joint association of ND2-237 Leu/Met polymorphism and coffee consumption with serum chloride levels among male Japanese health checkup examinees. For men with the ND2-237Leu genotype, higher coffee consumption may reduce the risk of low levels of serum chloride. Therefore, daily coffee intake is recommended for men with the ND2-237Leu genotype to reduce the risk of CVD. To the best of our knowledge, this is the first report of the effects of gene–diet interaction on serum chloride levels. These findings may contribute to individualized prevention strategies for CVD.

## Figures and Tables

**Table 1:** Clinical characteristics of the study participants by ND2-237 Leu/Met genotype.

	ND2-237Leu	ND2-237Met	*p* Value
	*N* = 245	*N* = 157	
Age (years) *	54.4 (7.8)	53.2 (7.8)	0.142
Body mass index (kg/m^2^) *	23.3 (2.8)	23.5 (2.6)	0.366
Systolic blood pressure (mmHg) *	125.8 (15.8)	125.7 (14.1)	0.934
Diastolic blood pressure (mmHg) ** welch	73.9 (10.6)	73.7 (9.1)	0.817
Low-density lipoprotein cholesterol (mg/dL) *	121.3 (34.4)	118.0 (30.8)	0.319
High-density lipoprotein cholesterol (mg/dL) ** welch	54.5 (13.5)	56.2 (16.1)	0.285
Triglyceride (mg/dL) ***	115 (84–160)	112 (84–158)	0.948
Uric acid (mg/dL) *	5.94 (1.24)	5.94 (1.22)	0.970
Serum sodium (mEq/L) *	140.3 (2.0)	140.1 (1.9)	0.200
Serum chloride (mEq/L) *	101.3 (2.5)	100.8 (2.2)	0.062
Serum potassium (mEq/L) *	4.19 (0.28)	4.18 (0.26)	0.712
Serum calcium (mEq/L) *	9.33 (0.37)	9.38 (0.38)	0.180
Coffee consumption (<1 cup per day/1–3 cups per day/≥4 cups per day) (%) ****	44.5/46.2/9.3	36.3/51.6/12.1	0.237
Current smokers (%) ****	41.7	40.8	0.852
Alcohol consumption (non- or ex-/occasionally/ daily) (%) ****	18.2/35.2/46.6	13.4/38.2/48.4	0.431
Green tea consumption (<1 cup per day/1–4 cups per day/≥5 cups per day) (%) ****	21.9/41.7/36.4	19.8/47.1/33.1	0.562
Antihypertensive (%) ****	19.4	13.4	0.115

Age, body mass index, systolic blood pressure, diastolic blood pressure, low-density lipoprotein cholesterol, high-density lipoprotein cholesterol, uric acid, serum sodium, serum chloride, serum potassium, serum calcium are expressed as means (standard deviation). Triglyceride is expressed as the median (interquartile range). * Student’s *t*-test, ** Welch’s *t*-test, *** Mann-Whitney test, **** chi-square test. All *p* values depict significant differences between ND2-237Leu and ND2-237Met.

**Table 2:** Serum electrolyte levels by coffee consumption status and ND2-237 Leu/Met genotype.

	Coffee Consumption			*p* for Trend
	<1 Cup Per Day	1–3 Cups Per Day	≥4 Cups Per Day	
ND2-237Leu	*N* = 109	*N* = 113	*N* = 23	
Serum sodium levels (mEq/L)	140.1 (0.2)	140.4 (0.2)	141.2 (0.4)	0.033
Serum sodium levels (mEq/L) ^†^	140.1 (0.2)	140.5 (0.2)	141.1 (0.4)	0.044
Serum chloride levels (mEq/L)	100.9 (0.2)	101.4 (0.2)	102.9 (0.5) **^,^***	0.001
Serum chloride levels (mEq/L) ^†^	100.7 (0.3)	101.4 (0.3)	102.4 (0.5) *	0.002
Serum potassium levels (mEq/L)	4.19 (0.03)	4.19 (0.03)	4.20 (0.05)	0.904
Serum potassium levels (mEq/L) ^†^	4.18 (0.03)	4.20 (0.03)	4.22 (0.06)	0.439
Serum calcium levels (mEq/L)	9.33 (0.04)	9.33 (0.04)	9.32 (0.08)	0.867
Serum calcium levels (mEq/L) ^†^	9.40 (0.04)	9.35 (0.04)	9.33 (0.08)	0.306
**ND2-237Met**	***N*** = **57**	***N*** = **81**	***N*** = **19**	
Serum sodium levels (mEq/L)	140.1 (0.2)	140.1 (0.2)	139.8 (0.4)	0.679
Serum sodium levels (mEq/L) ^†^	140.7 (0.3)	140.8 (0.3)	140.5 (0.5)	0.700
Serum chloride levels (mEq/L)	100.8 (0.3)	100.9 (0.2)	100.8 (0.5)	0.935
Serum chloride levels (mEq/L) ^†^	101.3 (0.4)	101.2 (0.3)	101.3 (0.5)	0.965
Serum potassium levels (mEq/L)	4.17 (0.03)	4.18 (0.03)	4.18 (0.06)	0.905
Serum potassium levels (mEq/L) ^†^	4.14 (0.05)	4.15 (0.04)	4.16 (0.07)	0.836
Serum calcium levels (mEq/L)	9.39 (0.05)	9.40 (0.04)	9.30 (0.09)	0.507
Serum calcium levels (mEq/L) ^†^	9.41 (0.07)	9.39 (0.06)	9.24 (0.10)	0.175

^†^ Serum sodium levels, ^†^ serum chloride levels, ^†^ serum potassium levels, and ^†^ serum calcium levels are expressed as least-square means (standard error) adjusted for age, body mass index, alcohol consumption, habitual smoking, green tea consumption, and antihypertensive medication. The Bonferroni correction for multiple comparisons was applied; * *p* < 0.05, ** *p* < 0.005 vs. <1 cup of coffee per day, *** *p* < 0.05 vs. 1–3 cups of coffee per day.

**Table 3:** Odds ratios (ORs) and 95% confidence intervals (CIs) for low levels of serum chloride (serum chloride levels <100 mEq/L) by ND2-237 Leu/Met genotype and coffee consumption.

Genotype and Coffee Consumption	Frequency (%)		OR (95% CI)	Adjusted OR ^†^ (95% CI)
	Normal Levels of Serum Chloride (Serum Chloride Levels ≥100 mEq/L)	Low Levels of Serum Chloride (Serum Chloride Levels <100 mEq/L)		
ND2-237Leu				
<1 cup per day	80 (73.4)	29 (26.6)	1 (reference)	1 (reference)
1–3 cups per day	89 (78.8)	24 (21.2)	0.744 (0.400–1.382)	0.615 (0.308–1.226)
≥4 cups per day	22 (95.7)	1 (4.3)	0.125 (0.016–0.973) *	0.096 (0.010–0.934) *
			*p* for trend = 0.032	*p* for trend = 0.028
ND2-237Met				
<1 cup per day	41 (71.9)	16 (28.1)	1 (reference)	1 (reference)
1–3 cups per day	60 (74.1)	21 (25.9)	0.897 (0.419–1.922)	0.803 (0.339–1.902)
≥4 cups per day	16 (84.2)	3 (15.8)	0.480 (0.123–1.875)	0.361 (0.076–1.718)
			*p* for trend = 0.353	*p* for trend = 0.264

^†^ OR adjusted for age, body mass index, habitual alcohol consumption, habitual smoking, green tea consumption, and antihypertensive medication. * *p* < 0.05.
